# Primary Liver Perivascular Epithelioid Cell Tumor (PEComa): Case Report and Literature Review

**DOI:** 10.3390/medicina60030409

**Published:** 2024-02-28

**Authors:** Mindaugas Kvietkauskas, Austeja Samuolyte, Rokas Rackauskas, Raminta Luksaite-Lukste, Gintare Karaliute, Vygante Maskoliunaite, Ruta Barbora Valkiuniene, Vitalijus Sokolovas, Kestutis Strupas

**Affiliations:** 1 Clinic of Gastroenterology, Nephrourology, and Surgery, Institute of Clinical Medicine, Faculty of Medicine, Vilnius University, M. K. Ciurlionio 21, 03101 Vilnius, Lithuania; 2 Department of Radiology, Nuclear Medicine and Medical Physics, Institute of Biomedical Sciences, Faculty of Medicine, Vilnius University, M. K. Ciurlionio 21, 03101 Vilnius, Lithuania; 3 Department of Pathology, Forensic Medicine and Pharmacology, Institute of Biomedical Sciences, Faculty of Medicine, Vilnius University, M. K. Ciurlionio 21, 03101 Vilnius, Lithuania

**Keywords:** liver, perivascular epithelioid cell tumor, PEComa, literature review

## Abstract

A primary liver perivascular epithelioid cell tumor (PEComa) is an extremely rare entity. In this article, we present a case report with a review of the literature on the patients diagnosed with primary liver PEComa and an elaboration of diagnostic and treatment modalities. A systematic literature search was conducted using the terms “perivascular epithelioid cell tumor”, “PEComa”, “liver”, and “hepatic”. All articles describing patients diagnosed with primary liver PEComa were included. We identified a total of 224 patients of primary liver PEComa from 75 articles and a case from the present study with a significant preponderance of females (ratio 4:1) and with a mean age of 45.3 ± 12.1 years. Most of the patients (114 out of 224, 50.9%) were asymptomatic. A total of 183 (81.3%) patients underwent surgical hepatic resection at the time of diagnosis, while 19 (8.4%) underwent surveillance. Recurrence and metastases were detected in seven (3.1%) and six (2.7%) patients, respectively. In conclusion, surgical resection remains the cornerstone of therapy; however, the presence of nonspecific imaging features makes it difficult to reach a definite diagnosis preoperatively. Therefore, a multidisciplinary approach should be the gold standard in selecting the treatment modality.

## 1. Introduction

Perivascular epithelioid cell tumors (PEComas) are a rare group of mesenchymal neoplasms composed of histologically and immunohistochemically distinctive perivascular epithelioid cells that were first proposed by Bonetti et al. in 1992 [1,2]. This group includes angiomyolipoma, lymphangioleiomyomatosis, clear-cell “sugar” tumors, clear-cell myomelanocytic tumors of the falciform ligament or ligamentum teres, and other tumors that are classified as PEComa–NOS (not otherwise specified) [3]. They have a perivascular distribution and express both melanocytic and smooth muscle markers [4]. Generally, PEComas are considered as benign tumors; however, the prediction of biological behavior is not clearly established. The most common locations of PEComas are the ovaries, uterus, gastrointestinal tract, bladder, abdominal wall, heart, and pancreas [5]. Cases of primary liver PEComas are extremely rare [6,7,8].

Surgical resection plays a crucial role in the treatment of this disease. However, correct preoperative diagnosis is rarely achieved due to the presence of unspecific radiological features; furthermore, the differential diagnosis with hepatocellular carcinoma is complicated and depends solely on morphology [4,9,10]. Until today, only a limited number of primary liver PEComa cases have been described worldwide. Due to the rarity and different sites of presentation, the management of these tumors is still a matter of debate in terms of the timing of surgery and the need for multimodal treatment [11].

In this article, we present a case report with a review of the literature of patients diagnosed with primary liver PEComa. We also intend to discuss and suggest possible diagnostic and treatment strategies.

## 2. Materials and Methods

A literature review was performed according to the guidelines from the Preferred Reporting Items for Systematic Reviews and Meta-analyses (PRISMA) [12]. The literature search was conducted independently by two authors to identify the English-written published articles (until December 2023) on primary liver PEComa. The PubMed data set was consulted matching the terms “perivascular epithelioid cell tumor”, “PEComa”, “liver”, and “hepatic” with “AND” and “OR”. The references of each article were assessed to complete the research.

Inclusion criteria: articles reporting primary liver PEComa, written in English, and papers with the longest follow-up or the largest sample size in the case of articles published by the same study group or based on the same data set. Exclusion criteria: not English-written and secondary liver PEComas.

From the eligible studies, two authors independently extracted data, including study characteristics (first author name and year), number of patients, general data (gender and age), course of the disease, diagnostic modalities, tumor characteristics, treatment, and outcomes. Disagreements between the authors were resolved by consensus; if no agreement could be reached, a third senior author made the decision.

Along with the cases reported in the literature, a patient with primary liver PEComa who recently underwent liver surgery at Vilnius University Hospital Santaros Klinikos (Vilnius, Lithuania) was also included in the analysis and is presented as a case report. Signed informed consent was obtained from this patient for any surgical and clinical procedure. Since this was a retrospective observational study with a review of the literature, formal consent for this study was not required and no approval of the institutional research committee was needed.

## 3. Case Report

**Figure 1:**
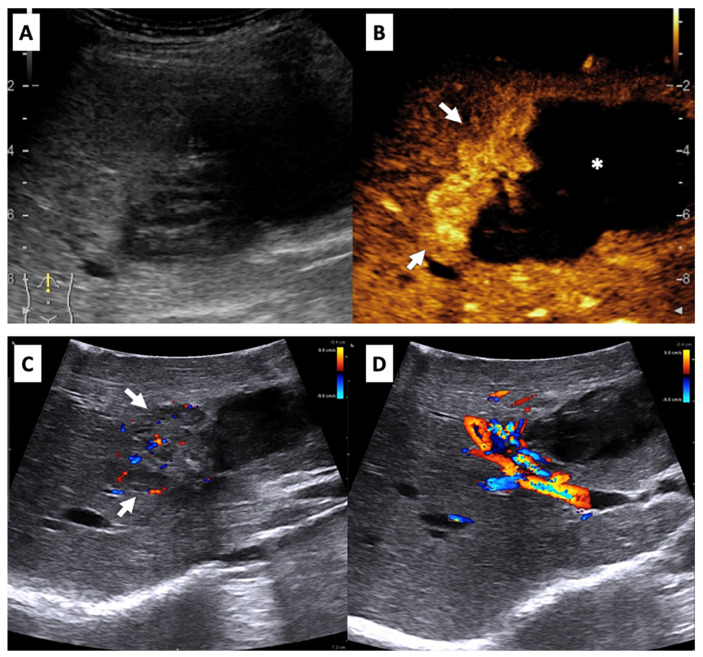
Images of preoperative contrast-enhanced and intraoperative ultrasound. Preoperative conventional (**A**) and contrast-enhanced ultrasound of the liver, arterial phase (B). A hypervascular ill-defined mass (arrows) in the right liver lobe (**B**), closely related to avascular heterogenous intraparenchymal liquid collection—hematoma (asterisk). Intraoperative ultrasound (**C**,**D**). A heteroechogenic vascularized solid mass in the right liver lobe (**C**), close to the main right anterior arterial and portal vascular branches (**D**).

A 42-year-old woman without any comorbidity was incidentally diagnosed with a mass lesion in liver S8 after a routine ultrasound (US) examination. Fine needle biopsy was performed in a small peripheral hospital. Histologically, the primary suspicion was a malignancy with epithelioid cell morphology, possibly non-hepatocytic origin, but full immunophenotyping was not performed and the final diagnosis was established after tumor resection. The post-intervention period was complicated by an asymptomatic liver hematoma that spontaneously ruptured after 19 days. An urgent laparotomy was performed and the hemoperitoneum was evacuated. The active hemorrhage within liver S4b was managed by placing temporary gauze tampons. For further treatment, the patient was referred to our center. The further course of the patient was uneventful. Two CT scans were performed three months apart without any change in dynamics. Four months after surgery, contrast-enhanced US revealed a hypervascular ill-defined 3.5 × 2.5 cm mass in S8 of the liver, closely related to avascular heterogenous intraparenchymal 8 × 6 cm liquid collection—hematoma in S4a and S4b (Figure 1A,B). Elective surgery was scheduled after a multidisciplinary team evaluation and the patient underwent a right hepatectomy with intraoperative US examination (Figure 1C,D). The operation and the postoperative course were uneventful, and the patient was discharged from the hospital 7 days after surgery.

**Figure 2:**
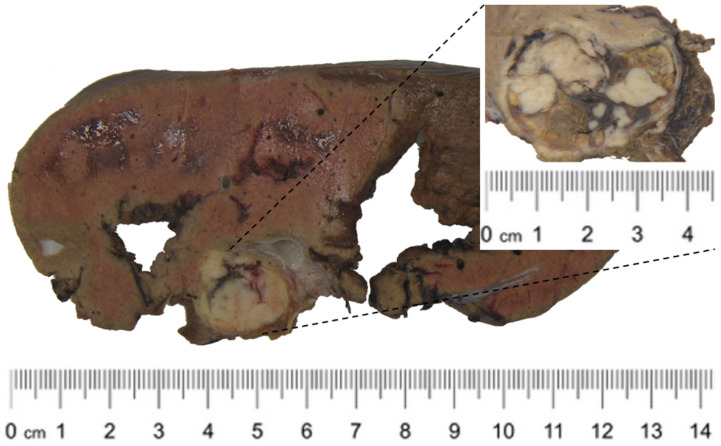
Macroscopic findings of the resected tissue specimen. The cut surface of the tumor shows a heterogeneous mass with a lobulated and pseudo-encapsulated appearance.

**Figure 3:**
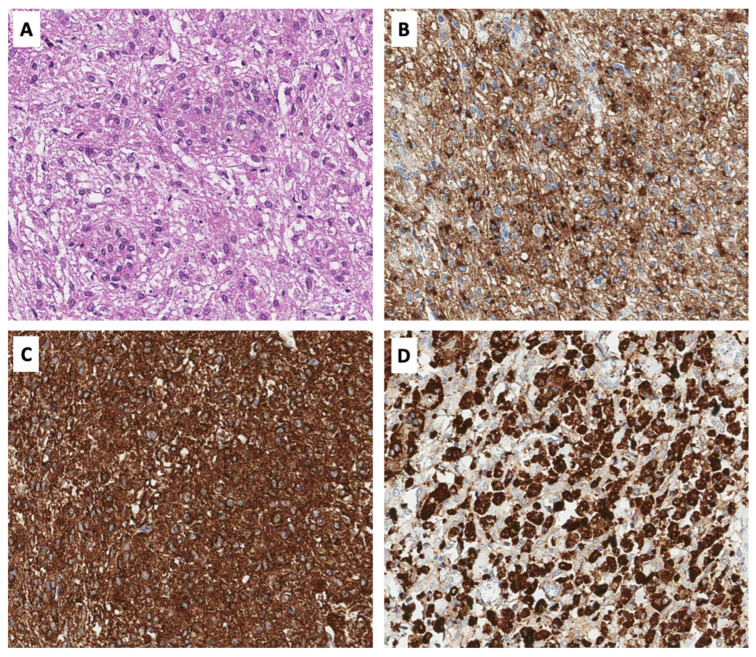
Histopathological and immunohistochemical features of primary liver PEComa case. Nested, lobular solid structures of monotonous spindle and epithelioid cells with eosinophilic or clear mildly granular cytoplasm (**A**). Tumor cells show positive cytoplasmic reaction for HMB-45 (**B**), SMA (**C**), and CD68 (**D**). Original magnification ×200.

Macroscopically, the specimen contained a 13.5 × 12 × 7 cm liver fragment with a 3.7 × 3 × 3 cm heterogeneous, lobulated, and pseudo-encapsulated tumor (Figure 2). Microscopically, the tumor was partially confined by a thin layer of fibrous tissue and had an expansive and infiltrative growth front. It was formed by nested, lobular, and solid structures of monotonous spindle and epithelioid cells with eosinophilic or clear finely granular cytoplasm and ovoid, polymorphic nuclei with low mitotic activity (1 mitosis/50 high-power fields) (Figure 3A). Examination revealed microscopically negative resection margins.

Immunohistochemistry showed strong and diffuse cytoplasmic reactions for human melanoma black 45 (HMB45), smooth muscle actin (SMA), H-caldesmon, and CD68 in the tumor cells (Figure 3B–D and Table 1). Desmin and Melan-A/MART1 (melanoma antigen recognized by T cells) were expressed in 60% and 30% of tumor cells, respectively. Only several cells were CD117-positive, and all were negative for microphthalmia transcription factor (MiFT), S100, transcription factor E3 (TFE3), and discovered on gastrointestinal stromal tumors protein 1 (DOG1). The Ki67 proliferation index was <1%. Overall, the tumor was diagnosed as a primary liver PEComa and classified as PEComa–NOS.

There is no clinical or radiographic evidence of recurrence or metastases 36 months after surgery.

## 4. Results of the Literature Review

**Figure 4:**
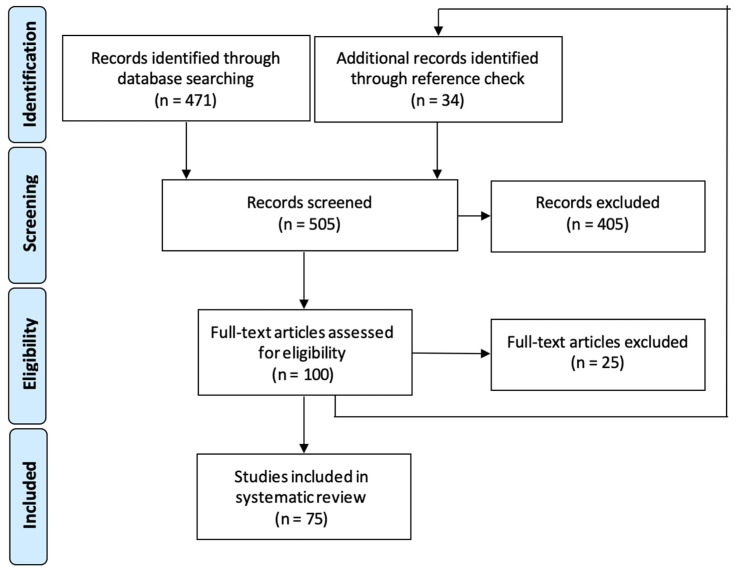
Flow chart of included studies in the literature review.

We identified 471 records from the database and 34 records through further reference checks (Figure 4). In total, 505 articles were screened by the title and the abstracts, following which 100 full-text articles were assessed for eligibility. We included 75 articles gathering data of 223 patients with primary liver PEComa. Including our case, a total of 224 patients with primary liver PEComa, aged 21–79 (median 47, mean 45.3 ± 12.1) years, were analyzed (Table 2 and Table S1). Most of the reports (46 out of 59, 61.3%) originated from centers in Asia and were single case reports (78.7%). Shan Zhang et al. [13] and Yang X et al. [14] published the largest case series, including 26 and 35 patients, respectively. In a complete cohort of patients, there was a significant preponderance of females (79.5%, 178 out of 224) with only 20.5% (46 out of 224) being male. A total of 114 out of 224 (50.9%) patients were asymptomatic, while 103 (46.0%) had symptoms, and for 7 (3.1%), data were unspecified. Out of 103 patients who presented with symptoms, the most common symptoms were abdominal pain (50.0%), abdominal discomfort (33.2%), nausea (6.6%), loss of appetite (6.6%), and weight loss (3.8%). A total of 165 (73.7%) patients underwent a computed tomography (CT) scan as the primary radiological imaging modality, 105 (46.9%) patients underwent magnetic resonance imaging (MRI), and 97 (43.3%) patients had an ultrasound (US). The majority of patients (94.2%, 211 out of 224) had a single lesion in the liver, while 5.8% (13 out of 224) had multiple lesions. A total of 126 (56.3%) tumors were located in the right lobe of the liver, 91 (40.6%) tumors in the left lobe, and 6 (2.7%) tumors in the caudate lobe (in 1 case, tumor localization was unspecified). The tumors’ size ranged from 1 to 30 cm in diameter (median 5.2, mean 7.1 cm). In 2014, Zhou et al. published a clinical case with the largest primary liver PEComa with a tumor diameter of 30 × 25 cm [15]. Pathology diagnosis before treatment was assessed in 45 (20.9%) of the patients by performing a biopsy, and out of them, PEComa was suspected in 29 (64.4%) patients. A total of 183 (81.3%) patients underwent surgical hepatic resection at the time of diagnosis, while 19 (8.4%) underwent surveillance and the data of 13 (5.8%) were unspecified. Three (1.3%) patients underwent tumor arterial embolization followed by microwave coagulation therapy or radiofrequency ablation. Neoadjuvant treatment was prescribed for three (1.3%) patients who had operations and four (1.8%) patients received adjuvant systemic treatment after surgery.

An immunohistochemical profile was performed for 196 patients. PEComas were identified to be strongly positive for HMB-45 (98.5%, *n* = 193/194), SMA (96.9%, *n* = 126/130), and Melan-A (98.7%, *n* = 155/157), while they were negative for S100 (35.9%, *n* = 28/78). Angiomyolipoma was the most common tumor type of the PEComa family (77.9%, *n* = 53) in the liver, while other tumors were less frequently observed: 10.3%—PEComa–NOS, 7.4%—clear-cell myomelanocytic tumor of the falciform ligament or *ligamentum teres*, 2.9%—lymphangioleiomyomatosis, and 1.5%—clear-cell “sugar” tumor.

The median follow-up period was 14 (3–108) months. Recurrence and metastases were detected in seven (3.1%) and six (2.7%) patients, respectively.

## 5. Discussion

This is a review of the literature on the management of patients with primary liver PEComas. We provide the representative patient profile of this rare disease. Moreover, this review consists of diagnostic and clinical treatment strategies and follow-up outcomes providing the highest level of data currently available.

The most described tumor of the PEComa group in the liver is angiomyolipoma. In our case, there was the lack of angiomyolipoma-specific histological features, such as adipocytic and prominent vascular components, and since there was no association with ligament structures, the tumor was classified as PEComa–NOS, which accounts for 10.3% of all reported cases in the literature.

The occurrence of PEComas can be associated with the female gender with a four times higher incidence rate; however, it was not explained and remains a matter of debate since the histopathogenesis remains uncertain. One hypothesis is that it may originate from undifferentiated neural crest cells with the capability of expressing the phenotype of melanocytes and smooth muscles, while a smooth muscle origin or a pericytic origin have been considered to be other possibilities [16]. The involvement of the TSC pathway in these tumors can suggest some possibilities: it has been previously proposed that B-raf activity in cells lacking TSC2 may play a role in cell differentiation; moreover, the TSC pathway negatively regulates the Wnt/beta-catenin pathway and beta-catenin regulates the transcription of genes involved in cell proliferation and differentiation. Nevertheless, a better understanding of PEComa’s histopathogenesis is warranted.

The majority of the patients are asymptomatic, although others can have non-specific symptoms such as abdominal pain, discomfort, nausea, or loss of appetite and/or weight. On clinical examination, a palpable abdominal mass could be the only sign of disease depending on tumor size and localization. There are no specific blood tests; however, widely used cancer markers such as α-fetoprotein, carbohydrate antigen 19-9 (CA 19-9), and carcinoembryonic antigen (CEA) must be performed for differential diagnosis of a liver mass.

Liver PEComas are difficult to diagnose due to their asymptomatic nature. Furthermore, a preoperative radiological imaging gold standard is lacking; therefore, a definite diagnosis is established after histological evaluation [17]. A single lesion is nine times more frequent than multiple, with preferential origin in the right liver lobe, reaching up to 30 cm in size. However, there is little knowledge of the underlying mechanism. One can speculate that more tumors originate in the right liver lobe due to higher blood flow with carcinogens from the gastrointestinal tract [18].

A CT scan is the most commonly used method of imaging due to its wide availability. CT imaging typically demonstrates a heterogeneous hypodense mass with either a well- or ill-defined margin, while in contrast-enhanced CT, only primary PEComas show marked enhancement in the arterial phase, while mild-to-moderate enhancement is shown in the equilibrium phase [10]. Previous reports have suggested that hypervascularity and arteriovenous characteristics in contrast-enhanced CT or MRI are a useful diagnostic feature of PEComa [8,19,20]. Contrast-enhanced US could demonstrate early-phase enhancement of the tumor and rapid drainage of the reagent to veins, suggesting a PEComa [21]. As in our case, contrast-enhanced US showed a hypervascular ill-defined mass in liver S8 with signs of rupture and hematoma.

Pathological evaluation is essential for the accurate diagnosis of PEComa, which is identified by distinct histological features of perivascular epithelioid cells and immunohistochemical positivity for melanocytic and smooth muscle markers, such as HMB45 and SMA, respectively [10]. In order to achieve this, fine-needle biopsy or laparoscopy may be needed [19]. However, awareness of this disease is warranted, since in 30%, a definitive diagnosis was not reached even after biopsy and must be always kept in mind by either the pathologist or clinician.

Currently, surgical resection is the gold standard method that has been used to treat this disease and can achieve a radical cure in most cases [22]. In 2014, Bergamo et al. treated a large and aggressive variant of PEComa with mTOR inhibitor in a neoadjuvant setting, since these tumors are mechanistically linked through the activation of the mTOR signaling pathway as mesenchymal neoplasms [11,23]. With this treatment, significant tumor shrinkage was achieved allowing for the radical removal of the mass without surgical complications or sequelae [11]. Thus, those patients with inoperable or borderline resectable tumors should be evaluated for systemic neoadjuvant treatment or arterial embolization to obtain tumor shrinkage and facilitate further surgical removal. However, a multidisciplinary approach is mandatory in determining the resectability of this disease, especially in the liver, where atypical and anatomical resection or even transplantation might be considered.

Initially, most PEComas exhibit benign behavior; however, there have been reports of local invasion and remote metastasis [9,20,24,25]. Folpe et al. reported an association between malignant clinical behavior and histopathological features and proposed criteria to define the tumor as malignant [26]. These criteria include tumor size greater than 5 cm, infiltrative growth pattern, high nuclear grade, high cellularity, necrosis, mitotic activity greater than 1 mitosis/50 high-power fields, and vascular invasion. Two or more criteria define the tumor as malignant. If only tumor size exceeds 5 cm or multinucleated giant cells are found, the malignant potential of the PEComa is considered as uncertain [3,26]. According to this assessment, our case fulfilled only one criterion of malignant PEComa: it had a partially infiltrative growth pattern. Other signs such as necrosis were considered as post-biopsy changes. Therefore, a benign course is more likely in our case. After 6 months of follow-up, no recurrence or metastasis was observed. Our literature review revealed a 3.1% rate of recurrence and a 2.7% rate of metastases after surgery, emphasizing the importance of patient follow-up and if needed additional adjuvant treatment in patients with high-risk features in order to increase disease control.

The present review is limited by its inability to pool the data for meta-analysis. Because most of the studies were single case reports or small case series, high-quality meta-analysis was not feasible for any of the outcomes measured. Despite this limitation, these findings remain meaningful for clinicians and bring additional awareness of this rare pathology.

## 6. Conclusions

Primary liver PEComa is a rare yet challenging pathology. It is associated with female gender with a four times higher incidence rate. As seen from the reviewed literature, the majority of patients will have non-specific symptoms such as abdominal pain, discomfort, nausea, or loss of appetite and/or weight. Clinical examination is also complicated as the only sign of disease during physical assessment could be a palpable abdominal mass. A preoperative radiological imaging gold standard is lacking, with a CT scan being the most commonly used method of imaging due to its availability; however, there are no specific imaging features. The prognosis varies and depends on histopathological risk factors with angiomyolipoma being the most common tumor type. Despite the lack of evidence, surgical resection remains the cornerstone of multimodal treatment as it can be seen from the literature review as well as the case report. Adjuvant treatment should be considered in high-risk patients. Adopting a multidisciplinary approach in the context of primary liver PEComas is crucial for the highest standard of care in treatment decision-making.

## Figures and Tables

**Table 1:** Immunohistochemical profile of primary liver PEComa case.

Marker	Positive Cells (%)
Melanocytic markers	
HMB45	100
Melan-A/MART1	30
MiTF	-
S100	-
TFE3	-
Smooth muscle markers	
SMA	100
Desmin	60
H-caldesmon	90
Other markers	
CD68	100
CD117	Several
DOG1	-
Ki67	<1

HMB45, human melanoma black 45; MART1, melanoma antigen recognized by T cells; MiFT, microphthalmia transcription factor; TFE3, transcription factor E3; SMA, smooth muscle actin; DOG1, discovered on gastrointestinal stromal tumors protein 1.

**Table 2:** Summary of the literature review data.

Parameter	Value
Patient characteristics	
Female sex, *n* (%)	178 (79.5%)
Mean age (years)	45.3
Continent of publications	
Asia, *n* (%)	46 (61.3%)
Europe, *n* (%)	19 (25.3%)
North America, *n* (%)	7 (9.3%)
South America, *n* (%)	1 (1.3%)
Africa, *n* (%)	1 (1.3%)
Australia, *n* (%)	1 (1.3%)
Manifestation	
Abdominal pain, *n* (%)	53 (50.0%)
Discomfort, *n* (%)	35 (33.0%)
Nausea, *n* (%)	7 (6.6%)
Tumor characteristics	
Average size (mm)	71.2
Min	13.0
Max	300.0
Single nodule, *n* (%)	211 (94.2%)
Multi-nodule, *n* (%)	13 (5.8%)
RL, *n* (%)	126 (56.0%)
LL, *n* (%)	91 (40.4%)
CL, *n* (%)	6 (2.7%)
Treatment modalities	
Biopsy, *n* (%)	45 (20.1%)
Surgery, *n* (%)	183 (81.3%)
Arterial chemoembolization, *n* (%)	3 (1.3%)
Adjuvant treatment, *n* (%)	4 (1.8%)
Neoadjuvant treatment, *n* (%)	3 (1.3%)
Surveillance, *n* (%)	19 (8.4%)
Immunohistochemistry	
HMB45, *n* (%)	193/193 (100.0%)
Melan-A, *n* (%)	156/158 (98.7%)
S100, *n* (%)	50/78 (64.1%)
SMA, *n* (%)	130/144 (90.3%)
Desmin, *n* (%)	40/67 (59.7%)
H-caldesmon, *n* (%)	2/5 (40.0%)

RL, right lower lobe; LL, left lower lobe; CL, caudate lobe.

## Data Availability

The data that support the findings of this study are available from the first author upon reasonable request.

## Supplementary Materials

The following are available online at https://www.mdpi.com/article/10.3390/medicina60030409/s1, Table S1: Published cases of primary liver PEComas. References [27,28,29,30,31,32,33,34,35,36,37,38,39,40,41,42,43,44,45,46,47,48,49,50,51,52,53,54,55,56,57,58,59,60,61,62,63,64,65,66,67,68,69,70,71,72,73,74,75,76,77,78,79,80,81] are cited in the supplementary materials.
