# Supplementary material for: Primary Liver Perivascular Epithelioid Cell Tumor (PEComa): Case Report and Literature Review
Source: Medicina (Kaunas). 2024 Feb 28;60(3):409. doi: 10.3390/medicina60030409 (PMC10972467; doi:10.3390/medicina60030409)
Supplement: Supplementary file 1 [file medicina-60-00409-s001.zip › medicina-2869266-supplementary.pdf]

Table S1. Published cases of primary liver PEComas.

	Authors	Year	Continent	Gender	Age, years	Symptoms	Tumor Size and Localization (US, CT, and/or MRI)	Pathology Diagnosis before Treatment	Initial Treatment	Outcomes
1	Dalle et al. [9]	2000	Europe	F	70	Abdominal pain, nausea and loss of appetite	Size: 15 cm Location: RLL	Biopsy	Percutaneous drainage of the liver abscess, trisegmentectomy 3 months after	After 5 months of follow-up, recurrence and multiple metastases in the residual liver
2	Yamasaki et al. [27]	2000	Asia	F	30	Asymptomatic	Size: 3.2 × 3.0 cm Location: RLL	No	Partial hepatectomy	After 1 year of follow-up, no recurrence or metastases
3	Tryggvason et al. [28]	2004	Europe	F	42	Abdominal pain and change in bowel habits	Size: 7 cm Location: LLL	No	Left lateral segmentectomy	After 23 months of follow-up, no recurrence or metastases
4	Parfitt et al. [20]	2006	Europe	F	60	Abdominal pain and tenderness	Size: 14 × 11 cm Location: RLL	Biopsy	Right hemihepatectomy	After 9 years of follow-up, metastases to multiple sites
5	Larbcharoensub et al. [29]	2007	Asia	F	31	Abdominal pain	Size: 1.8 × 1.6 cm Location: S8	No	Partial hepatectomy with cholecystectomy	After 6 months of follow-up, no recurrence or metastases
6	Fang et al. [8]	2007	Asia	F	56	Asymptomatic	Size: 5.1 × 4.2 cm Location: S4	No	Surgery (N/A)	After 2 years of follow-up, no recurrence or metastases
				F	63	Abdominal pain	Size: N/A Location: S1	No	Surgery (N/A)	After 1 year of follow-up, no recurrence or metastases
7	Della Vigna et al. [30]	2008	Europe	F	46	Asymptomatic	Size: 5.3 cm Location: S3	Biopsy	Left lateral segmentectomy	N/A

8	Paiva et al. [31]	2008	Europe	F	51	Abdominal pain	Size: N/A<br>Location: N/A	No	Surgery (N/A)	After 25 months of follow-up, no recurrence or metastases
9	Strelczyk et al. [32]	2009	Europe	F	57	Abdominal pain, nausea, vomiting, epigastric fullness	Size: 20 × 18 cm<br>Location: RLL	No	Right hemihepatectomy with cholecystectomy	After 53 months of follow-up, no recurrence or metastases
10	Priola et al. [33]	2009	Europe	F	36	Acute abdominal distension with severe epigastric and mesogastric pain	Size: 11 cm<br>Location: LLL	No	Left hemihepatectomy	After 34 months of follow-up, no recurrence or metastases
11	Akitake et al. [21]	2009	Asia	F	36	Asymptomatic	Size: 3.5 × 3.5 cm<br>Location: S2	No	Lateral segmentectomy	After 18 months of follow-up, no recurrence or metastases
12	Ahn and Hur [34]	2011	Asia	F	36	Asymptomatic	Size: 7.0 × 5.6 cm<br>Location: LLL	No	Lateral segmentectomy	After 3 months of follow-up, no recurrence or metastases
13	Selvaggi et al. [35]	2011	Europe	F	42	Dyspnea, temperature, abdominal discomfort and weight loss	Size: 7 cm<br>Location: S5/8	No	Urgent explorative laparotomy, hemostasis and biopsy	Died after 25 days
14	Xie et al. [36]	2011	North America	F	32	Fever	Size: <4 cm (>10 lesions)<br>Location: RLL and LLL	Biopsy	Surveillance	N/A
15	Liu et al. [37]	2012	Asia	4 F<br>1 M	26–57	3 Asymptomatic<br>2 Symptomatic (N/A)	Size: Largest 16 × 6 cm<br>Location: N/A	No	3 partial hepatectomies,<br>1 left lobectomy and partial right hepatectomy, 1 partial hepatectomy and partial nephrectomy	After 29–101 months of follow-up, no recurrence or metastases

16	Tan and Xiao [25]	2012	Asia	6 F<br>1 M	33–75 (mean 48.7)	3 Abdominal pain<br>4 Asymptomatic	Size: 2.5–8.5 cm (mean 4)<br>Location: 5 RLL, 1 LLL, 1 RLL and LLL	No	N/A	After 1–4 years of follow-up, 2 were malignant
17	Yu and Tang [38]	2013	Asia	F	41	Abdominal pain	Size: 1.9 × 1.9 cm<br>Location: S6	No	Right hepatectomy	After 9 months of follow-up, no recurrence or metastases
18	Zhao et al. [39]	2013	Asia	M	58	Abdominal pain	Size: 7.6 × 5.2 cm<br>Location: RLL	No	Right S4 segmentectomy	N/A
19	Shen et al. [19]	2013	Asia	M	55	Asymptomatic	Size: 1.5 × 1.6 cm<br>Location: S6	No	Surgery (N/A)	After 12 months of follow-up, no recurrence or metastases
20	Khaja et al. [40]	2013	North America	F	51	Symptomatic (N/A)	Size: N/A<br>Location: RLL	Biopsy	N/A	N/A
21	Patra et al. [41]	2013	Asia	F	50	Abdominal pain, vomiting, loss of appetite	Size: 15 × 12 cm and 10 × 10cm<br>Location: S5/6 and S8	No	Surgery (N/A)	After 24 months of follow-up, no recurrence or metastases
22	Jafari et al. [42]	2013	Europe	F	53	Abdominal pain	Size: 7.5 × 5.5 cm<br>Location: S2, S3	Biopsy	Left lateral bisegmentectomy	After 14 months of follow-up, no recurrence or metastases
23	Cheung et al. [43]	2013	Asia	F	53	Abdominal pain	Size: 10 cm<br>Location: RLL	No	Right hepatectomy	After 3 months of follow-up, no recurrence or metastases
24	Tay et al. [44]	2013	Asia	F	51	Loss of appetite and weight loss	Size: 9 × 8.8 cm<br>Location: S2/3	No	S2 and S3 bisegmentectomy	After 9 months of follow-up, no recurrence or metastases
25	Ameurtesse et al. [45]	2014	Africa	F	63	Abdominal pain, fatigue, and weight loss	Size: 6.4 × 8 cm<br>Location: S4	No	Central segmentectomy	After 9 months of follow-up, no recurrence or metastases

26	Bergamo et al. [11]	2014	Europe	F	31	Vomiting and gastric reflux	Size: N/A<br>Location: RLL	Biopsy		Neoadjuvant sirolimus followed by resection of S4b, S5 and S6, and adjuvant sirolimus	N/A
27	Zhou et al. [15]	2014	Asia	F	34	Abdominal discomfort	Size: 30 × 25 cm<br>Location: LLL	No		Left hepatectomy, cholecystectomy	After 71 months of follow-up, no recurrence or metastases
28	Liu et al. [1]	2014	Asia	F	25	Asymptomatic	Size: 1.8 × 1.5 cm<br>Location: S7	No		Partial hepatectomy	After 12 months of follow-up, no recurrence or metastases
29	Khan et al. [46]	2014	North America	M	61	Asymptomatic	Size: 6.7 × 6.5 cm<br>Location: S7	Biopsy		S7 and S8 bisegmentectomy	N/A
30	Abhirup et al. [47]	2015	Asia	F	72	Abdominal pain	Size: 10 × 8 cm<br>Location: S8	No		Extended right hepatectomy	Recurrence after 8 months
31	Maebayashi et al. [17]	2015	Asia	M	58	Abdominal bloating	Size: 4.5 cm<br>Location: S3	No		S3 partial segmentectomy	After 5 years of follow-up, no recurrence or metastases
32	Wang et al. [22]	2015	Asia	F	29	Asymptomatic	Size: 15.5 × 14.2 cm<br>Location: S5/6	No		S5 segmentectomy and S6 partial segmentectomy with cholecystectomy	N/A
33	Kiriyama et al. [7]	2016	Asia	M	47	Low-back pain	Size: 1.8 × 0.5 cm<br>Location: LLL	No		Left hemihepatectomy	After 56 months of follow-up, no recurrence or metastases
34	Hao et al. [48]	2016	Asia	F	51	Asymptomatic	Size: 8 cm<br>Location: S6	No		Partial hepatectomy and selective hepatic artery ligation	After 3 months of follow-up, recurrence and second operation
				F	30	Asymptomatic	Size: 2.5 cm<br>Location: S8	No		Partial hepatectomy	After 8 months of follow-up, no recurrence or metastases

				M	25	Asymptomatic	Size: 8 cm<br>Location: S6	No	Radical hepatectomy	After 3 years of follow-up, no recurrence or metastases
35	Tang et al. [49]	2016	Asia	F	32	Abdominal pain	Size: 6.5 × 6 cm<br>Location: S5	No	S5 segmentectomy	After 9 months of follow-up, no recurrence or metastases
36	Lan and Hua [50]	2016	Asia	F	40	Asymptomatic	Size: 9.4 × 6.5, 4.5 × 5, 2.5 × 2.3 cm<br>Location: S4, S5, S6	Biopsy	Surgery (N/A)	After 1 year of follow-up, no recurrence or metastases
37	Schaeffer and Poulin [51]	2016	North America	F	49	Abdominal pain and diabetes mellitus	Size: 2.9 cm<br>Location: S8	No	Partial hepatectomy	N/A
38	O'Malley et al. [24]	2017	North America	16 F<br>4 M	35–77 (mean 53)	18 Asymptomatic<br>2 Epigastric discomfort	Size: 1.3–15 cm (mean 5.1)<br>Location: 11 RLL and 9 LLL	13 Biopsy<br>7 No	3 Surgery<br>15 Surveillance<br>2 N/A	After follow-up: 5 underwent surgery (1 urgently for bleeding), 2 had radiofrequency ablations, 8 remain on surveillance, and 2 developed metastatic disease after initial surgery (at 9 months and 2 years)
39	Son et al. [52]	2017	Asia	F	56	Asymptomatic	Size: 3.2 × 3 cm<br>Location: S5	No	Partial hepatectomy	After 8 months of follow-up, no recurrence or metastases
40	Hekimoglu and Haberal [5]	2017	Asia	F	79	Abdominal pain and tenderness	Size: 5.2 × 4.3 cm<br>Location: S8 and S1	Biopsy	Partial hepatectomy	After 6 months of follow-up, no recurrence or metastases
41	Chen et al. [53]	2017	Asia	6 F<br>1 M	35–77 (median 43)	2 Abdominal pain<br>1 Emaciation	Size: <5 cm<br>Location: 3 LLL, 3 RLL and 1 S1	2 Biopsy	6 Partial hepatectomy	After 10–20 months of follow-up, no

						1 Lymphadenectasis<br>4 Asymptomatic			1 Tumor arterial embolization followed by microwave coagulation therapy	recurrence or metastases
42	Guan et al. [54]	2017	Asia	F	40	Asymptomatic	Size: 7.5 × 5 cm<br>Location: S8	Biopsy	Transarterial embolization followed by radiofrequency ablation	After 6 months of follow-up, no recurrence or metastases
43	Han et al. [10]	2017	Asia	F	36	Abdominal distention, cramps, and fever	Size: 3.7 × 2.4 cm<br>Location: S8	Biopsy	N/A	N/A
44	Cardoso et al. [55]	2017	Europe	F	37	Asymptomatic	Size: 1.6–2.9 cm (4 lesions)<br>Location: S2, S4	No	Left hepatectomy	After 6 months of follow-up, no recurrence or metastases
45	Altolagirre et al. [56]	2017	Europe	F	46	N/A	Size: 2.4 cm<br>Location: S2	Biopsy	Limited resection (N/A)	After 6 years of follow-up, no recurrence or metastases
				F	45	N/A	Size: 2.5 cm<br>Location: S8	Biopsy	Limited resection (N/A)	After 4 years of follow-up, no recurrence or metastases
				F	48	N/A	Size: 7 cm<br>Location: Hilum	Biopsy	S1 segmentectomy	After 18 months of follow-up, no recurrence or metastases
				M	53	General symptoms (N/A)	Size: 3 cm<br>Location: LLL	Biopsy	Left hepatectomy	After 11 months of follow-up, no recurrence or metastases
46	Kirste et al. [6]	2018	Europe	F	52	Abdominal pain	Size: 1280 cm3<br>Location: S4	Biopsy	Neoadjuvant stereotactic body radiotherapy followed by extended	After 21 months of follow-up, no recurrence or metastases

									hemihepatectomy (S1, S4-8)	
47	Ma et al. [57]	2018	Asia	13 F	22–72 (median 35)	9 Asymptomatic<br>4 Abdominal pain<br>1 Loss of appetite	Size: 1–19.8 cm (median 3.8)<br>Location: 7 RLL, 4 LLL, 1 RLL and LLL, 1 Hepatic portal	No	Partial hepatectomy	After 2–60 months of follow-up, 9 cases exhibited no recurrence or metastases, and 4 cases were lost to follow-up
48	Dezman et al. [58]	2018	Europe	F	24	Abdominal pain	Size: 2 cm<br>Location: S4	Biopsy	Non-anatomical liver resection (N/A)	N/A
49	Kirnap et al. [59]	2018	Asia	F	22	Asymptomatic	Size: 17 × 15 cm<br>Location: LLL	No	Surgical resection with cholecystectomy	After 10 months of follow-up, no recurrence or metastases
50	Voulgari et al. [60]	2018	Europe	M	47	N/A	Size: 7 cm<br>Location: S1/2	No	Left lobectomy with S1 segmentectomy and cholecystectomy	After 18 months of follow-up, no recurrence or metastases
51	Lopes et al. [61]	2019	South America	F	55	Abdominal fullness	Size: 4 cm<br>Location: S3	No	Laparoscopic resection (N/A)	N/A
52	Kiuchi et al. [62]	2019	Asia	F	46	N/A	Size: 14 and 1 cm<br>Location: LLL and RLL	No	Left and partial right hepatectomy	N/A
53	Nie et al. [16]	2019	Asia	14 F<br>8 M	23–76 (mean 47.1)	17 Asymptomatic<br>3 Abdominal pain<br>2 Discomfort of upper abdomen	Size: 1.4–23.6 (mean 7.67) cm<br>Location: 13 RLL, 3 RLL and 2 S1	21 No<br>1 Biopsy	21 Surgery<br>1 Surveillance	After 2–98 months of follow-up, 11 cases showed no recurrence or metastases, 10 were lost to follow-up, and 1 had a stable lesion
54	Li et al. [63]	2019	Asia	F	46	N/A	Size: 2 × 1.5 cm<br>Location: S1	No	Laparoscopic S1 segmentectomy	After 8 months of follow-up, no recurrence or metastases

				F	50	N/A	Size: 1.8 × 1.3 cm<br>Location: S1	No	Laparoscopic S1 segmentectomy	After 8 months of follow-up, no recurrence or metastases
55	Chai et al. [64]	2020	Australia	F	62	Asymptomatic	Size: 2.5 × 1.6 cm<br>Location: S5	No	Right hemihepatectomy	N/A
				F	51	Asymptomatic	Size: 4.6 and 1 cm<br>Location: S7 and S8	No	S7 and S8 bisegmentectomy	N/A
56	Xu et al. [65]	2020	Asia	F	53	Fever	Size: N/A<br>Location: RRL	No	Combined multisegmental hepatectomy (N/A)	After 14 months of follow-up, no recurrence or metastases
57	Lopez et al. [66]	2020	Europe	F	29	Asymptomatic	Size: 1.8 cm<br>Location: S2	No	S2 segmentectomy	N/A
				M	27	N/A	Size: 3 × 2.2 cm<br>Location: S6	No	S6 segmentectomy	N/A
58	Nakra et al. [67]	2020	Asia	F	42	Asymptomatic	Size: 5 × 3, 1.5 and 1.5 cm<br>Location: S4a, S2 and S6	Biopsy	Left hepatectomy	N/A
59	Case from present study	2020	Europe	F	42	Asymptomatic	Size: 3.5 × 2.5 cm<br>Location: S4a/4b/8	Biopsy	Right hepatectomy	After 6 months of follow-up, no recurrence or metastases
60	Carlo De la Sancha MD [68]	2021	North America	F	38	Right upper quadrant pain, nausea, and diarrhea	Size: 12 cm<br>Location:<br>RL	Biopsy	N/A	N/A
61	Mochizuki K et al. [69]	2021	Asia	F	31	Asymptomatic	Size: 0,13 cm<br>Location: anterior superior segment	No	Minimally invasive laparoscopic resection using ICG-FI	N/A
62	Shan Zhang et al. [13]	2021	Asia	17 F<br>9 M	26-77 (median 50)	Non-specific (nausea, vomiting, loss of appetite, or weight loss)	Size: 0.5-13.0 cm (mean 6.5 cm)<br>Location:<br>LL 11<br>RL 13<br>CL 2	No	16 hepatic lobe resection<br>8 tumor resection<br>2 received mTOR sirolimus	After 25-99 months of follow up, favorable prognosis in 24 patients, 1 reoperation for liver cancer, 1

											hepatic pain after discharge
63	Huang et al. [70]	2021	Asia	M	30	Asymptomatic	Size: 4.6×3.6 cm<br>Location:<br>CL	No		Laparoscopic hepatic caudate lobe resection	After 7 months of follow-up, no tumor recurrence was observed
64	Jisheng Zhu et al. [71]	2022	Asia	10 F<br>2 M	37-66 (median 49)	9 had no symptoms, 2 had abdominal pain and 1 had abdominal discomfort	Size: 2.3-16.0 cm (mean 6.5 cm)<br>Location: S1, four S3, S4b, two in S6, three in S2, S7, three in S5, and S8.	No		4 left lateral sectionectomies<br>1 segmentectomy<br>1 extended left hemipatectomy + caudate lobectomy<br>1 partial hepatectomy	Median follow-up was 23,5 (range 5.6–95.5) months. No tumor recurrence or metastasis were observed, and all patients were alive
65	Yang X et al. [14]	2022	Asia	29 F<br>6 M	21-75 (mean 48,0)	13 patients had upper abdominal pain or discomfort, 22 had no symptoms	Size: 1.2 -12.0 cm (mean 5.1), 2 patients with multiple tumors<br>Location:<br>20 RL, 13 LL, 2 CL	No		Every patient accepted a partial hepatectomy	Average follow-up was 66.5 months (3-132 months). Two patients experienced tumor recurrence and one patient died of cardiovascular disease
66	Wang S et al. [72]	2022	Asia	F	50	Upper abdominal tenderness, chest pain.	Size: 5.0×3.5 cm<br>Location: LLL	No		Laparoscopic resection	After 24 months of follow-up, no evidence of tumor recurrence or metastasis
67	Chai J et al. [73]	2023	Asia	F	33	Abdominal pain	Size: 15 cm.<br>Location: Right posterior lobe	No		Partial hepatectomy	No recurrence or metastasis (follow-up time unknown)
68	Okamoto T et al. [74]	2023	Asia	F	66	Asymptomatic	Size: 1.8 × 2.0 × 1.7 cm<br>Location: tumor adjacent to the cystic duct	EUS-guided fine needle aspiration		Tumor resection without common bile duct resection	After 72 months of follow-up, no recurrence has been observed

69	Cai X et al. [75]	2023	Asia	F	51	Upper abdominal pain, fatigue, poor appetite	Size: 4.0 × 5.7 cm + multiple small lesions. Location: intrahepatic	Biopsy		Transcatheter arterial chemoembolization (for multiple tumors)	Follow up every 3 months showed little changes in lesions and the largest tumor (follow-up time unknown)
70	Matrood S et al. [76]	2023	Europe	F	51	Asymptomatic	Size: 2 cm Location: RL	Biopsy		Surveillance	After 3 years of follow up, no changes were observed
71	Velasco-Albendea FJ et al. [77]	2023	Europe	F	35	Lack of appetite, weight loss, asthenia, general malaise, and digestive discomfort	Size: 7.8 × 9.3 × 6.7 cm Location: RL	Biopsy		Right lobe hepatectomy	N/A
72	Kou YQ et al. [78]	2023	Asia	M	37	Abdominal pain for 1 month	Size: 2.5 × 2.0 × 2.3 cm Location: S6	No		Partial hepatectomy	After 12 months of follow-up, no recurrence or metastases
				F	70	Asymptomatic	Size: 5.0 × 3.0 × 3.1 cm Location: S3, S4.	No		Partial hepatectomy	After 35 months of follow-up, no recurrence or metastases
				F	30	Abdominal bloating	5.6 × 4.7 × 5.0 cm: Location: S8	No		Partial hepatectomy	After 46 months of follow-up, no recurrence or metastases
73	Dhaliwal K et al. [79]	2023	North America	M	59	Asymptomatic	Size: 2.2 × 2.0 cm Location: RL	Biopsy		Surveillance	Died after 3 months
74	Harwal R et al. [80]	2023	Asia	F	27	Asymptomatic	Size: 10 × 10 × 13 cm Location: LL	No		Right hepatectomy	After 1-year follow-up, no recurrence or metastasis

75	Yazıcı C et al. [81]	2023	Asia	F	41	Abdominal pain	Size: 3.5 × 3.0 cm Location: RL	No	Surgical resection	After 66 months of follow-up, no recurrence or metastasis.
